# Indications and Technique for Thoracic Segmental Spinal Anesthesia in Clinical Practice: A Narrative Review

**DOI:** 10.7759/cureus.84118

**Published:** 2025-05-14

**Authors:** Imran Ahmed Khan, Nurul Haque Siddiqui, Srikrishna S Ramachandra, Abhijit Nair

**Affiliations:** 1 Community Medicine, KMC Medical College and Hospital, Maharajganj, IND; 2 Anesthesiology, Balrampur Hospital, Lucknow, IND; 3 Anesthesiology, Ibra Hospital, Ibra, OMN

**Keywords:** acute postoperative pain, `anesthesia, regional anesthesia, subarachnoid block, thoracic segmental spinal anesthesia

## Abstract

Thoracic segmental spinal anesthesia (TSSA) represents an advanced technique in present clinical anesthetic practice. By lowering the systemic effects of anesthesia, preserving improved hemodynamic stability, and accelerating postoperative recovery, its targeted approach provides significant benefits in certain upper abdominal and thoracic procedures, particularly in high-risk patients. However, careful patient selection, exact technical execution, and in-depth understanding of thoracic anatomy are necessary for its successful implementation. Despite its advantages, TSSA is not yet widely adopted. The major concerns remain related to its technical complexity, principally the risk of spinal cord injury, and the necessity for specialized training and imaging guidance during needle placement. In recent years, there has been a surge in the number of articles describing the safety of TSSA for varying surgeries. This review aims to summarize various indications and the techniques described in the literature related to TSSA.

## Introduction and background

Spinal anesthesia (SA) or subarachnoid block is a common regional anesthesia technique introduced in the late 19th century. Traditionally, lumbar spinal anesthesia (LSA) has been the preferred technique due to its well-documented safety profile and widespread applicability across various surgical procedures [1]. However, there has been growing interest in thoracic segmental spinal anesthesia (TSSA) in recent years. This technique involves administering intrathecal local anesthetics at thoracic vertebral levels to achieve segmental blockade [2]. Unlike LSA, which often results in a more extensive sympathetic block and associated hemodynamic instability, particularly in patients with compromised cardiovascular reserve, TSSA offers a more targeted approach with better hemodynamic stability and reduced side effects [3].

TSSA has emerged as an alternative to LSA and general anesthesia (GA) for several surgical procedures. The technique has been refined with the advent of low-dose intrathecal anesthetics (isobaric and hypobaric local anesthetics)and improved imaging modalities, like ultrasonography, ensuring safer administration [4]. By limiting the spread of the anesthetic agent to specific thoracic dermatomes, TSSA minimizes the impact on lower limb motor function and preserves sympathetic tone, thereby reducing the risk of hypotension and bradycardia [5]. Additionally, TSSA has been associated with decreased opioid consumption, reduced postoperative nausea and vomiting, and faster recovery times compared to GA and LSA [6].

Despite its potential advantages, TSSA remains underutilized in routine clinical practice, primarily due to concerns regarding its safety and the technical expertise required for its administration [7]. However, emerging evidence suggests that TSSA is not only feasible but also beneficial across multiple surgical specialties, including orthopedic, abdominal, thoracic, and obstetric surgeries [3,8]. Given the expanding body of literature on TSSA, a comprehensive review is warranted to evaluate its varied clinical indications, identify areas where it offers superior outcomes, and highlight potential challenges in its implementation. This narrative review aims to evaluate and synthesize current evidence regarding the clinical indications for TSSA across different surgical specialties. By systematically analyzing the literature, we seek to provide insights into its technique and applicability, ultimately guiding anesthesiologists in optimizing its use in modern anesthesia practice.

## Review

Methods

A broad search strategy was used to incorporate the maximum relevant articles.

Search Strategy

A comprehensive literature search was conducted in databases and search engines including PubMed/MEDLINE, Embase, Scopus, and Google Scholar. Search terms and Boolean strategy were applied using primary keywords “thoracic segmental spinal anesthesia” OR segmental thoracic spinal anesthesia”, OR “thoracic spinal anesthesia”, AND “segmental spinal anesthesia”.

Eligibility Criteria

The study inclusion criteria were decided beforehand and presented according to the PICO (Population, Intervention, Comparison, Outcome) framework (Table 1).

**Table 1:** PICO framework of the study TSSA: thoracic segmental spinal anesthesia; PICO: Population, Intervention, Comparison, Outcome; LSA: lumbar spinal anesthesia

Component	Details
Population (P)	Patients undergoing surgical procedures where TSSA was used as an anesthetic technique (e.g., general surgery, laparoscopic surgery, cesarean section, orthopedic surgery, urological surgery, and thoracic surgery)
Intervention (I)	TSSA
Comparison (C)	Other anesthesia techniques (e.g., LSA, epidural anesthesia, and general anesthesia) or no direct comparison in some cases
Outcome (O)	Varied clinical indications and applications of TSSA, including feasibility, safety, efficacy, and advantages over other techniques

Inclusion Criteria

Studies reporting clinical indications of TSSA, consisting of randomized controlled trials (RCTs), comparative studies, observational studies, case reports, and case series, published in the English language. In comparative studies, only those data were included that reported TSSA participants.

Exclusion Criteria

Studies on LSA and GA without specific TSSA data were excluded. Any type of review article, editorials, expert opinions, and animal studies were also excluded.

Study Selection and Data Extraction

Two independent investigators (IAK and KS) screened titles and abstracts from databases PubMed, Medline, Scopus, Embase, and Google Scholar search engine till January 2025 using predefined keywords and Boolean operators ("Thoracic segmental spinal anesthesia" OR "segmental spinal anesthesia" OR "segmental thoracic spinal anesthesia” OR “thoracic spinal anesthesia” OR "spinal anesthesia in thoracic region"). Full-text reviews for eligible studies were conducted by the same two investigators. Disagreements between investigators were resolved through discussion, and in cases where consensus could not be reached, a third reviewer (AN) was consulted. Full-text articles were retrieved for the studies shortlisted based on the eligibility criteria. Data extraction included author, country, year of publication, study design, sample size, level of spinal block, LA and adjuvants used, surgical procedure, comorbidities, and clinical complications.

Results

**Figure 1:**
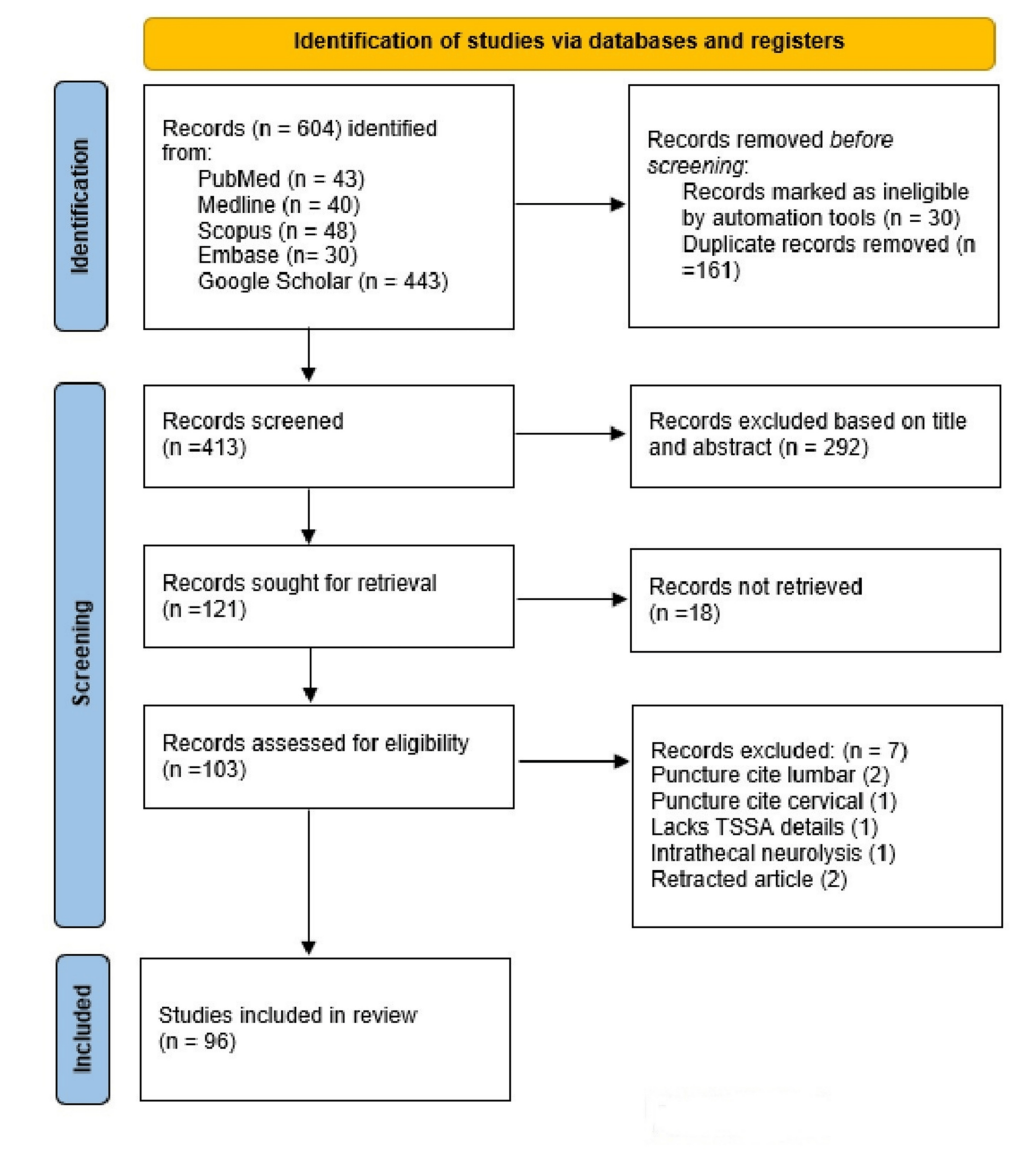
PRISMA flow diagram TSSA: thoracic segmental spinal anesthesia

A total of 604 potentially relevant articles were identified from PubMed (43), Medline (40), Embase (30), Scopus (48), and Google Scholar (443). After removing 30 records marked as ineligible by automation tools and 161 duplicate articles, 413 were available for title and abstract screening. The screening through titles and abstracts further removed 292 articles. Out of the remaining 121, the full text of 18 articles was not retrieved and was removed. The full text of the remaining 103 articles was retrieved and assessed, and based on the selection criteria, seven articles were excluded at this stage. Finally, the remaining 96 articles were selected for the review presented in Figure 1.

**Figure 2:**
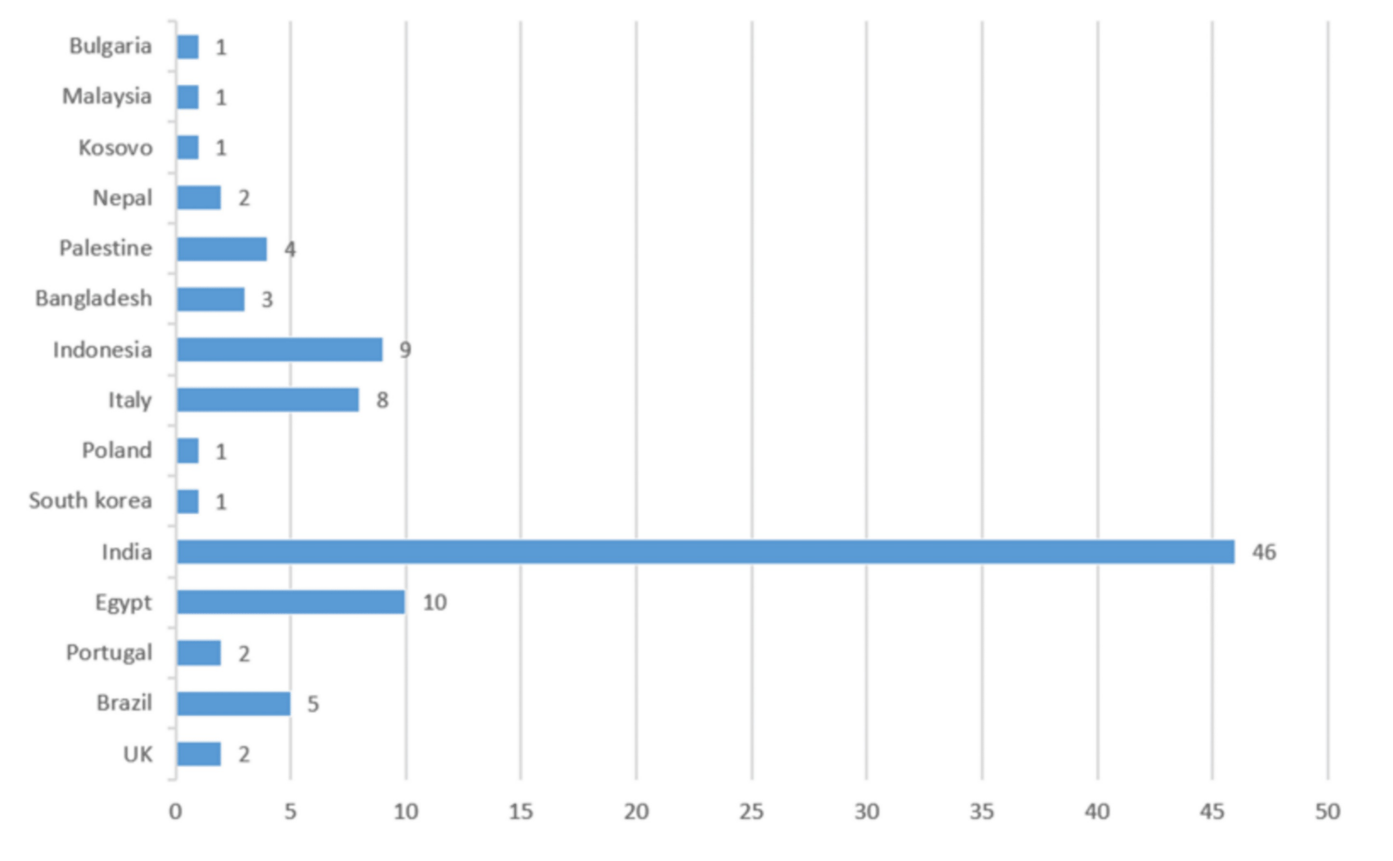
Country-wise publication on TSSA TSSA: thoracic segmental spinal anesthesia

A total of 6270 patients from 15 countries were included in 96 studies. A maximum of 46 articles were published from India (Figure 2).

**Figure 3:**
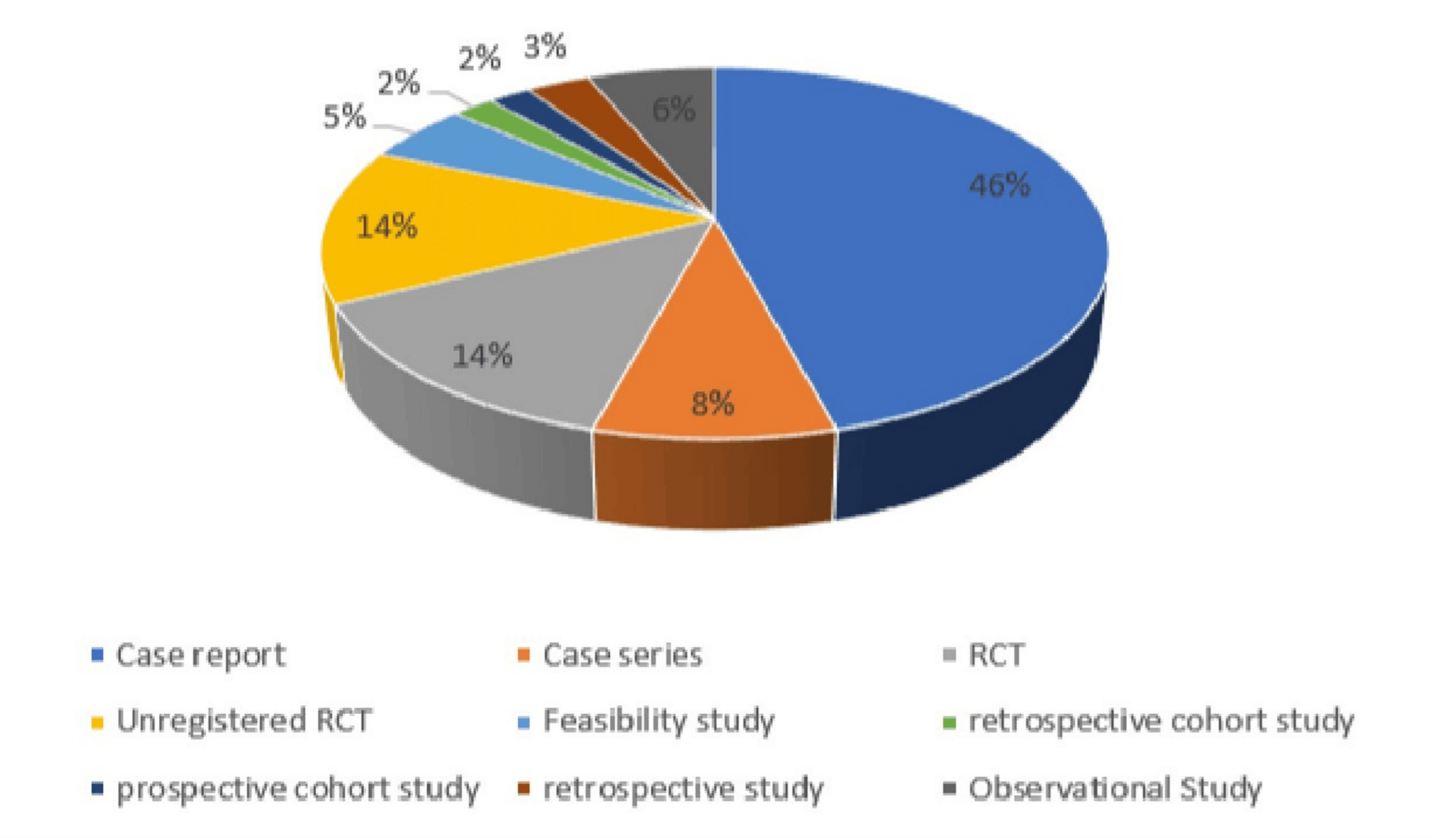
Categories of publications RCT: randomized controlled trial

The most common study type was a case report (Figure 3). There are 13 RCTs and an additional 13 unregistered RCTs. In most studies, comparisons of TSSA were done with GA and LSA.

**Figure 4:**
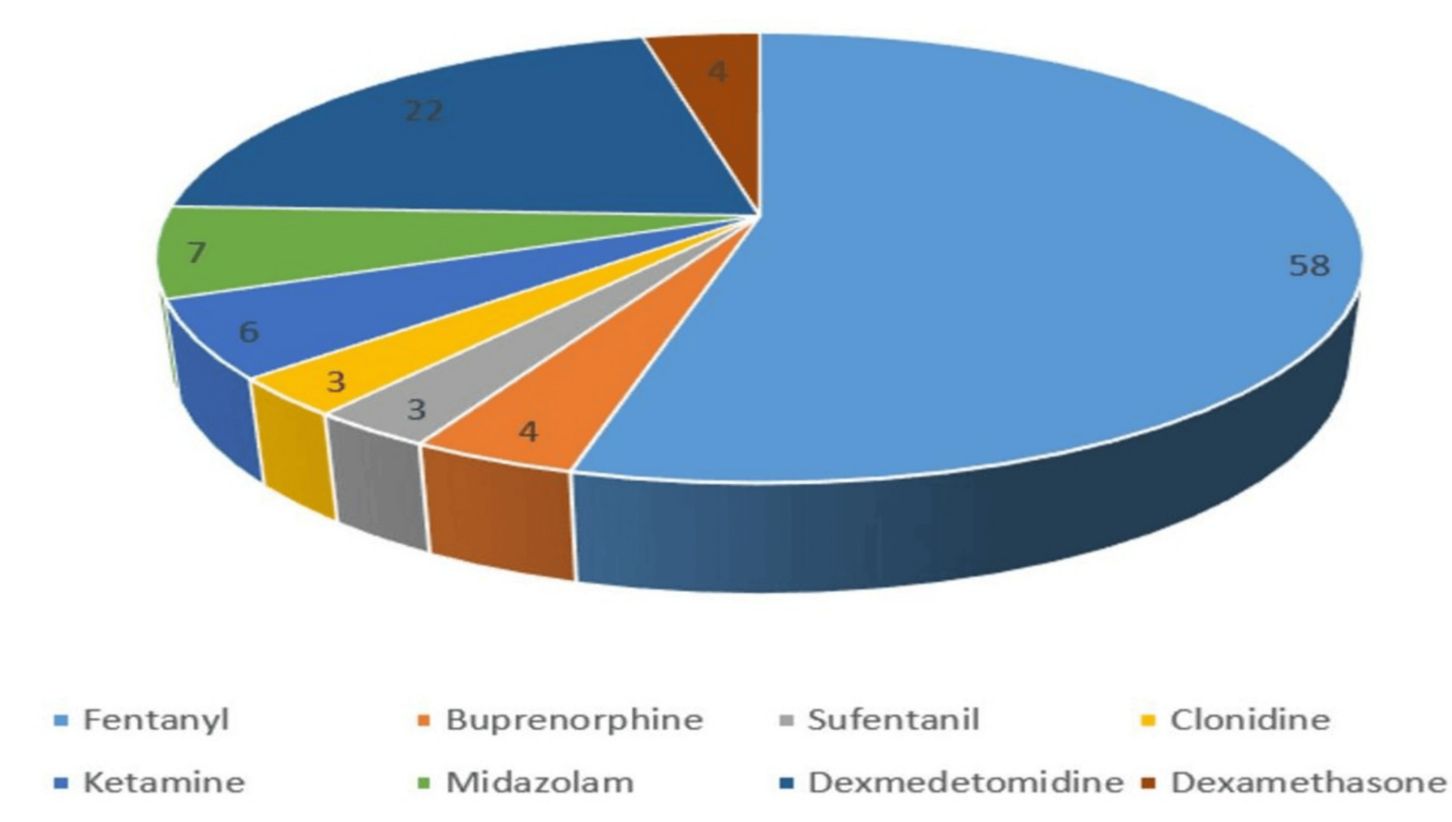
Intrathecal adjuvants used with local anesthetic

TSSA was given in isolation in 77 (80%) studies, and in 14 (14.6%) cases, it was given as CSE. One case reported ESPB with TSSA, and four cases were continuous TSSA. Needle sizes 25 and 27 were the most commonly reported spinal needles in 32 (33.3%) studies, each followed by size 26 in 14 (14.6%) studies. Bupivacaine, levobupivacaine, and ropivacaine with different baricities were used during TSSA. Two studies reported the use of hypobaric local anesthetics. The most common adjuvants were fentanyl (59 studies) and dexmedetomidine (19 studies). Other adjuvants were sufentanil, clonidine, buprenorphine, dexamethasone, and morphine. Ketamine and midazolam were used for intrathecal sedation in two studies. Adjuvants details were not provided in 11 studies (Figure 4).

**Figure 5:**
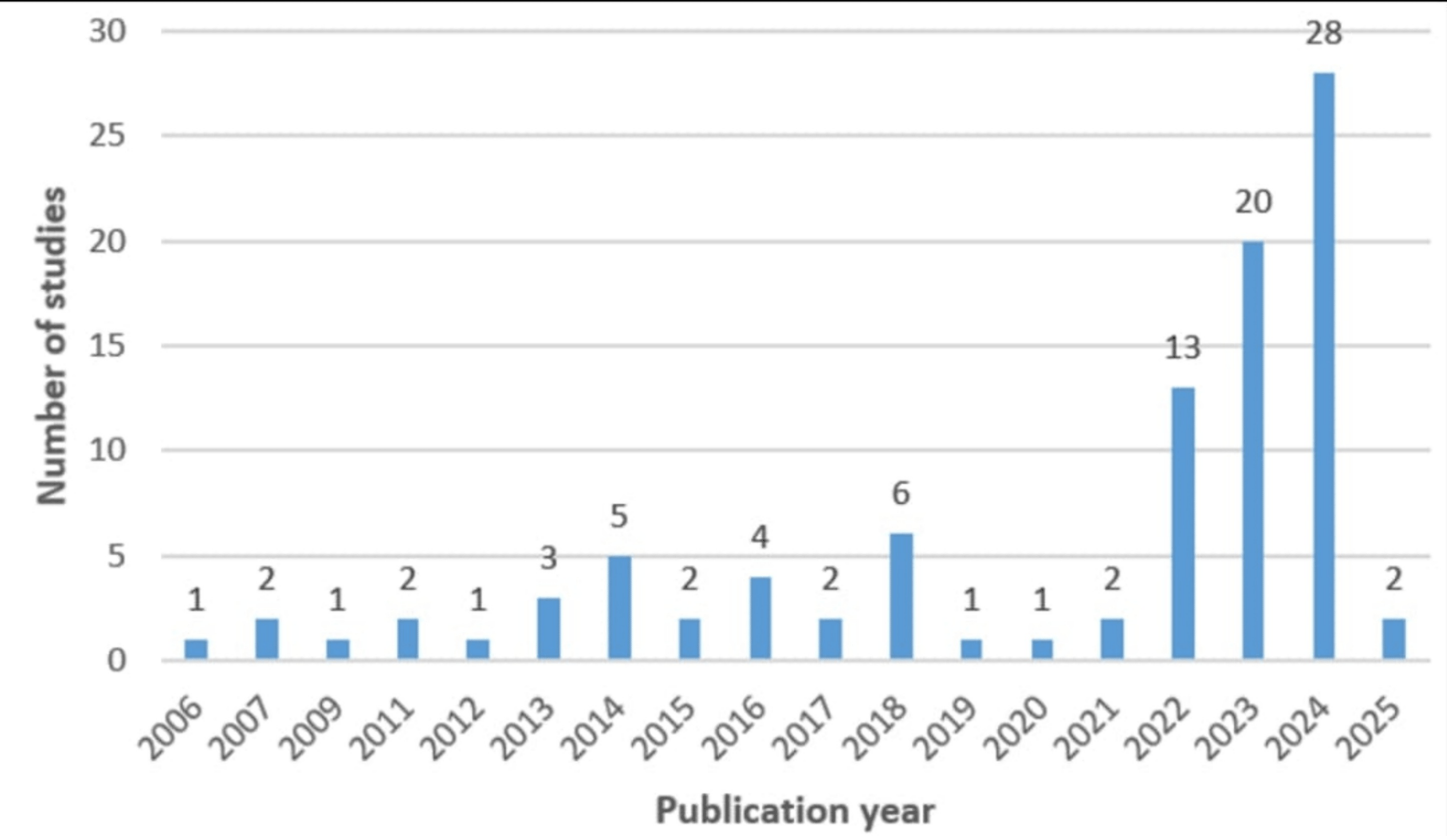
Number of publications related to TSSA over the years TSSA: thoracic segmental spinal anesthesia

Figure 5 showcases the number of publications related to TSSA over the years.

A total of 36 articles reported Laparoscopic surgeries, including 30 laparoscopic cholecystectomies. Other major indications were upper and lower abdominal surgeries (25 studies) and breast surgeries (19 studies) where TSSA was used. Some other operative procedures where TSSA was used included laparoscopic and open gynecological, PCNL, nephrectomy, laparotomy, lipoma, thoracoscopy, kyphoplasty, and upper extremity surgeries. Diabetes mellitus (DM), hypertension, chronic obstructive pulmonary disease (COPD), chronic kidney disease (CKD), and ischemic heart disease (IHD) were the comorbidities in which TSSA was used for operative procedures. Several studies also reported the use of TSSA in ASA-PS I and II patients (Table 2).

**Table 2:** Comorbidities among patients who received TSSA DCM: dilated cardiomyopathy, IHD: ischemic heart disease, CHF: congestive heart failure, ASD: atrial septal defect, COPD: chronic obstructive pulmonary disease, DM: diabetes mellitus, ILD: interstitial lung disease,

Comorbidities	Studies (number)
Cardiovascular	Hypertension, DCM, pacemaker, apical hypokinesia, IHD, CHF, heart transplant recipient, situs inversus, and ASD
Pulmonary	COPD, asthma, byssinosis, postpneumonectomy, ILD, and bronchiectasis
Endocrine	DM, obesity, and hyperthyroidism
Others	Coagulopathy, amyotrophic lateral sclerosis, squamous cell carcinoma, multiple comorbidities, Parkinson’s disease, pre-eclampsia, electrolyte imbalance, spinal deformities or previous spine surgeries, anemia, and Birt-Hogg-Dube syndrome

Table 3 summarizes the various types of surgery in which TSSA was used.

**Table 3:** Summary of various surgeries in which TSSA was used

Indications	Number of studies (%)
Laparoscopic surgery	36 (37.5)
Abdominal surgery	25 (26.0)
Breast surgery	19 (19.8)
Thoracotomy and thoracoscopic surgery	3 (3.1)
Incisional and inguinal hernia	3 (3.1)
Orthopedic surgery	3 (3.1)
Chest wall surgery	4 (4.2)
Spine surgery	3 (3.1)

Discussion

This study provides a comprehensive review of TSSA and highlights the growing clinical interest and expanding utility of TSSA across a wide spectrum of surgical specialties. The findings suggest that TSSA is not only a feasible and effective anesthetic technique but also offers distinct advantages over traditional LSA and GA, particularly in terms of hemodynamic stability, reduced opioid consumption, and expedited recovery [9-11]. Despite the concerns regarding technical expertise and safety, the inclusion of 13 RCTs [12-24] and 13 unregistered RCTs [9,25-36], along with numerous observational studies and case reports, reinforces the growing acceptance and reliability of TSSA in clinical practice.

While laparoscopic cholecystectomy and breast surgeries remain the most commonly reported applications, emerging indications include thoracoscopic procedures, kyphoplasty, nephrectomy, and even select upper extremity and gynecological surgeries [24,36]. The feasibility of TSSA in patients with significant comorbidities, such as DM, hypertension, CKD, and IHD, further suggests its potential as a preferred anesthetic technique in high-risk surgical populations [11,31,32].

The review highlights a growing trend of utilizing TSSA beyond conventional domains. TSSA was successfully used in a post-pneumonectomy patient for laparoscopic cholecystectomy [16]. Laparoscopic cholecystectomy was performed under TSSA in a high-risk patient with a cardiac pacemaker, dilated cardiac myopathy, and an ejection fraction of 20% [37]. Continuous TSSA with intrathecal midazolam, ketamine, and fentanyl was used in elderly patients having multiple comorbidities for conducting major abdominal surgeries [38]. The use of TSSA was also documented in a patient with Situs Inversus Totalis and Birt-Hogg-Dube syndrome [39,40].

Additionally, the evolving use of adjuvants and hypobaric solutions opens avenues for customized segmental blockade suited to specific surgical needs [39,41-103]. Future applications may extend to incorporate TSSA to enhanced recovery after surgery (ERAS) protocols, ambulatory surgical settings, and elderly or frail patients where minimizing hemodynamic fluctuations is crucial.

Despite the encouraging findings, several limitations in the current evidence base should be acknowledged. A significant proportion of studies are case reports and small observational studies, limiting the generalizability of results. Only a limited number of RCTs exist, and many comparative studies lack uniformity in methodology, drug dosing, and outcome measures. Furthermore, heterogeneity in patient populations, surgical procedures, and anesthetic techniques makes it challenging to draw definitive conclusions. There is also a lack of long-term safety data and standardized protocols for TSSA administration. Lastly, publication bias and underreporting of complications in smaller studies may skew the perceived safety profile. Robust, multicentric trials with standardized outcome reporting are essential to validate the routine use of TSSA in broader clinical practice.

## Conclusions

This review underscores the expanding role of TSSA as an effective and versatile technique across a wide range of surgical procedures. TSSA offers notable advantages over traditional anesthesia methods, including accepted hemodynamic stability, reduced opioid use, and faster recovery. Its successful application in patients with comorbidities and various surgical specialties, supported by evidence from case reports/series and observational studies, highlights its growing indications in clinical practice. With appropriate expertise and careful patient selection, TSSA has the potential to be a valuable addition to modern anesthesia protocols.

## References

[1] Balavenkatasubramanian Balavenkatasubramanian, Senthilkumar Senthilkumar, Kumar V (2023). Current indications for spinal anesthesia-a narrative review. Best Pract Res Clin Anaesthesiol.

[2] Khan IA, Ansari MA (2023). Thoracic segmental anesthesia: a new paradigm in anesthesiology practice. Arch Anesth Crit Care.

[3] le Roux JJ, Wakabayashi K, Jooma Z (2023). Defining the role of thoracic spinal anaesthesia in the 21st century: a narrative review. Br J Anaesth.

[4] Hamdi T, Mastari ES, Lubis AP, Ghozali I, Kemalasari N, Harahap AT (2025). Effectiveness and safety of thoracic segmental spinal anesthesia for breast surgery: a systematic review and meta-analysis. Narra J.

[5] Khan I, Paliwal N, Ahmad S (2022). Safety and feasibility of segmental thoracic spinal anaesthesia (STSA): a scoping review. Sch J App Med Sci.

[6] Gautam SN, Yadav R, Khanal A, Dangol S (2024). The safety and feasibility of segmental thoracic spinal anesthesia above umbilicus and breast surgery. Nepal Med Coll J.

[7] Khan IA, Paliwal NW (2025). Challenges in implementing thoracic segmental spinal anesthesia in routine anesthesia practice. SBV J Basic Clin Appl Health Sci.

[8] Kurdi MS, Agrawal P, Thakkar P, Arora D, Barde SM, Eswaran K (2023). Recent advancements in regional anaesthesia. Indian J Anaesth.

[9] Abdelmonem A (20111). Low dose hyperbaric bupivicaine injected at T12-L1 provides adequate anesthesia with stable hemodynamics for elderly patients undergoing TURP. Egypt J Anaesth.

[10] Imbelloni LE, Sant'anna R, Fornasari M, Fialho JC (2011). Laparoscopic cholecystectomy under spinal anesthesia: comparative study between conventional-dose and low-dose hyperbaric bupivacaine. Local Reg Anesth.

[11] Imbelloni LE (2014). Spinal anesthesia for laparoscopic cholecystectomy: thoracic vs. lumbar technique. Saudi J Anaesth.

[12] Imbelloni LE, Gouveia MA (2014). A comparison of thoracic spinal anesthesia with low-dose isobaric and low-dose hyperbaric bupivacaine for orthopedic surgery: a randomized controlled trial. Anesth Essays Res.

[13] Ellakany MH (2014). Thoracic spinal anesthesia is safe for patients undergoing abdominal cancer surgery. Anesth Essays Res.

[14] ELdeen HM (2016). Ultrasound guided pectoral nerve blockade versus thoracic spinal blockade for conservative breast surgery in cancer breast: A randomized controlled trial. Egypt J Anaesth.

[15] Mazy A, El-Domiaty A, Mageed NA, Motawi AA, Messeha M (2022). Comparison between thoracic paravertebral block and segmental thoracic spinal anesthesia in breast cancer surgery. Ain-Shams J Anesthesiol.

[16] Haq HU, Raghunath SV, Sushma V (2022). A comparison of general anesthesia and segmental thoracic spinalanesthesia regarding hemodynamic and respiratory stability Forlaparoscopic cholecystectomy. IAR J Med Surg Res.

[17] Paliwal N, Maurya N, Suthar OP, Janweja S (2022). Segmental thoracic spinal anesthesia versus general anesthesia for breast cancer surgery: a prospective randomized-controlled open-label trial. J Anaesthesiol Clin Pharmacol.

[18] Singhal G, Choudhary S, Saxena AK (2023). Comparison of thoracic segmental spinal anaesthesia and lumbar spinal anaesthesia for percutaneous nephrolithotomy. Int J Res Med Sci.

[19] Elzohry AA, Hegab AS, Khalifa OY, Elhossieny KM, Abdel Hameed FA (2023). Safety and efficacy of ultrasound-guided combined segmental thoracic spinal epidural anesthesia in abdominal surgeries and laparoscopic procedures: a prospective randomized clinical study. Anesth Pain Med.

[20] Verma AK, Kumar N, Srinivas C, Sahu P (2024). Comparison of the effectiveness and safety of segmental thoracic spinal anesthesia using isobaric levobupivacaine 0.5% versus hyperbaric levobupivacaine 0.5% in performing laparoscopic cholecystectomy: a prospective randomized controlled trial. Cureus.

[21] Singhal G, Kaur P (2024). Comparison of hemodynamic changes in thoracic segmental spinal anaesthesia and general anaesthesia for laparoscopic cholecystectomy. Indian J Clin Anaesth.

[22] Karthik GS, Srinivasan R, Sudheer R, Amabareesha M, Monisha TS, Kumar MD (2024). Thoracic spinal anaesthesia - An effective alternative to general anaesthesia in breast surgeries: a randomised, non-blinded study. Indian J Anaesth.

[23] Sangadala P, Talawar P, Tripathy DK, Kaushal A, Gupta A, Raj N (2024). Comparison of block characteristics and outcomes in opioid-free and opioid-based thoracic continuous spinal anaesthesia in patients undergoing major abdominal surgery: a double-blinded randomised controlled trial. Indian J Anaesth.

[24] Karthik GS, Chandra M, Sudheer R, Shwetha AH (2025). Combined thoracic segmental spinal anesthesia and erector spinae plane block in high-risk patients undergoing thoracoscopic surgery: a case series. Saudi J Anaesth.

[25] van Zundert AA, Stultiens G, Jakimowicz JJ, Peek D, van der Ham WG, Korsten HH, Wildsmith JA (2007). Laparoscopic cholecystectomy under segmental thoracic spinal anaesthesia: a feasibility study. Br J Anaesth.

[26] Elakany MH, Abdelhamid SA (2013). Segmental thoracic spinal has advantages over general anesthesia for breast cancer surgery. Anesth Essays Res.

[27] Ellakany M (2013). Comparative study between general and thoracic spinal anesthesia for laparoscopic cholecystectomy. Egypt J Anaesth.

[28] Kour L, Rasool A (2017). Comparison of low dose bupivacaine and ropivacaine in low thoracic combined spinal epidural anaesthesia for laparoscopic cholecystectomy. JK Science.

[29] Ahmed AM, Ali H, Helal O, Sobhi T (2017). Comparative study between continuous thoracic epidural vs. thoracic spinal anaesthesia in breast surgery. J Pain Relief.

[30] Hobaika AB, Silva CH, Alves NG, Isoni NC (20151). Two cases of thoracic spinal anaesthesia in patients with severe diseases. Egypt J Anaesth.

[31] Mehta N, Gupta S, Sharma A, Dar MR (2015). Thoracic combined spinal epidural anesthesia for laparoscopic cholecystectomy in a geriatric patient with ischemic heart disease and renal insufficiency. Local Reg Anesth.

[32] Daszkiewicz A, Copik M, Misiolek H (2016). Thoracic combined spinal-epidural anesthesia for laparoscopic cholecystectomy in an obese patient with asthma and multiple drug allergies: a case report. Innov Surg Sci.

[33] Bhalavi M, Shakya ML, Dandotiya D (2023). Comparison of two different doses of hyperbaric bupivacaine in segmental spinal anesthesia for laparoscopic cholecystectomy. Asian J Pharm Clin Res.

[34] Deshpande N, Agarwal K, Hatgaonkar R, Paliwal N (2023). Efficacy of thoracic segmental spinal anaesthesia along with unilateral erector spinae block in patients undergoing unilateral modified radical mastectomy and axillary dissection: A novel multicentric study. Asian J Pharm Clin.

[35] Kumbhare N, Dubey A, Mishra CS, Yadav V, Chakravarty N (2023). To compare isobaric thoracic spinal anaesthesia versus general anaesthesia in laparascopy cholecystectomy. Int J Pure Med Res.

[36] Manasra MR, Heih OQ, Adwan RF, Maraqa MA (2024). The use of thoracic segmental spinal anaesthesia for thoracoscopic diaphragmatic hernia repair in an adult with cardiac compromise. Cureus.

[37] Mehta N, Gupta KC, Sharma S, Dar MR (2016). Thoracic combined spinal epidural anesthesia in patient of dilated cardiomyopathy undergoing laparoscopic cholecystectomy. J Anaesthesiol Clin Pharmacol.

[38] Vincenzi P, Starnari R, Faloia L (2020). Continuous thoracic spinal anesthesia with local anesthetic plus midazolam and ketamine is superior to local anesthetic plus fentanyl in major abdominal surgery. Surg Open Sci.

[39] Deshpande J, Jacob ME (2023). Fibroadenoma excision under thoracic segmental spinal anaesthesia in isolated situs inversus totalis: a case report. Arch anesthesiol crit care.

[40] Ghosh TK, Roy P (2023). Thoracic segmental spinal anaesthesia for laparoscopic cholecystectomy in a case of birt-hogg-dube syndrome. Asian J Med Sci.

[41] Ghozali I, Hamdi T, Yusmaidi Y, Sitepu JF (2022). Single-shot thoracic spinal anesthesia (TSA) in pediatric patient under laparoscopic cholecystectomy: a case report. J Soc Med.

[42] Vincenzi P, Stronati M, Garelli P, Gaudenzi D, Boccoli G, Starnari R (2023). Segmental thoracic spinal anesthesia for laparoscopic cholecystectomy with the “hypobaric” technique: a case series. Local Reg Anesth.

[43] van Zundert AA, Stultiens G, Jakimowicz JJ, van den Borne BE, van der Ham WG, Wildsmith JA (2006). Segmental spinal anaesthesia for cholecystectomy in a patient with severe lung disease. Br J Anaesth.

[44] Hobaika AB (2007). Thoracic spinal anesthesia for gastrostomy in a patient with severe lung disease. Acta Anaesthesiol Scand.

[45] Imbelloni LE, Fornasari M, Fialho JC (2009). Combined spinal epidural anesthesia during colon surgery in a high-risk patient: case report. Rev Bras Anestesiol.

[46] Patel K, Salgaonkar S (2012). Segmental thoracic spinal anesthesia in patient with byssinosis undergoing nephrectomy. Anesth Essays Res.

[47] Kim BS, Joo SH, Joh JH, Yi JW (2013). Laparoscopic cholecystectomy in patients with anesthetic problems. World J Gastroenterol.

[48] Mahmoud AA, Hussein HA, Girgis K, Kamal AM, Nafady HA (20141). The novel use of spinal anesthesia at the mid-thoracic level: a feasibility study. Egypt J Cardiothorac Anesth.

[49] Paria R, Surroy S, Majumder M, Paria B (2014). Spinal at T10 (10th thoracic). IOSR-JDMS.

[50] Mehta N, Dar MR, Sharma S, Mehta KS (2016). Thoracic combined spinal epidural anesthesia for laparoscopic cholecystectomy: a feasibility study. J Anaesthesiol Clin Pharmacol.

[51] Kejriwal AK, Begum S, Krishan G, Agrawal R (2017). Laparoscopic cholecystectomy under segmental thoracic spinal anesthesia: a feasible economical alternative. Anesth Essays Res.

[52] Castellani D, Starnari R, Faloia L (2018). Radical cystectomy in frail octogenarians in thoracic continuous spinal anesthesia and analgesia: a pilot study. Ther Adv Urol.

[53] El Moutaz H, Wahab HA, Ghany JM (2018). Comparative study of segmental thoracic spinal versus thoracic epidural anesthesia for laparoscopic cholecystectomy. Ann Med.

[54] Kour L, Gupta KC (2018). Comparison of effect of isobaric bupivacaine vs hyperbaric bupivacaine on haemodynamic variables in thoracic combined spinal epidural anaesthesia for laparoscopic cholecystectomies. Int J Res Med Sci.

[55] Kour L, Gupta KC, Mehta N, Mehta KS (2018). Laparoscopic cholecystectomy under low thoracic combined spinal epidural anaesthesia: a comparative study between isobaric and hyperbaric bupivacaine. IOSR-JDMS.

[56] Parthasarathy S, Arthi PR (2018). Administration of segmental thoracic spinal anaesthesia for excision of lipoma back of chest - a case report. Br J Pharm Med Res.

[57] Cotugno M, Dallaglio M, Cantadori L (2020). Right open nephrectomy under combined spinal and peridural operative anesthesia and analgesia (CSE): a new anesthetic approach in abdominal surgery. Arch Ital Urol Androl.

[58] Fitrisyah A, Zainal R, Darwis E (2021). Laparoscopic cholecystectomy under segmental thoracic spinal anesthesia. Arch Med Case Rep.

[59] Chauhan R, Sabharwal P, Sarna R, Meena S (2021). Thoracic spinal anesthesia for cesarean section in severe pre-eclampsia: exploring a new dimension. Ain-Shams J Anesthesiol.

[60] Atmawan DB, Kurniawan HA, Priyambada P (2022). Thoracic spinal anaesthesia for modified radical mastectomy (MRM). J Anaesth Pain.

[61] Suryo C, Widana IW (20221). Thoracal segmental spinal anesthesia for lower back lipoma excision. MEDICINUS.

[62] Arora G (2022). Breast surgery under thoracic spinal anesthesia - a case report. Int J Anesthesiol Res.

[63] Khan MK, Uddin MA, Uddin MA, Rab WR (2022). Thoracic segmental spinal anesthesia in patient with severe COPD in open cholecystectomy. Cent Med Coll J.

[64] Imbelloni LE, Fornasari M, Ant’Anna Filho RGB (2022). Thoracic spinal anesthesia is safe and without neurological sequelae: study with 1,406 patients. Int J Anesth Anesthesiol.

[65] Vincenzi P, Stronati M, Isidori P, Iuorio S, Gaudenzi D, Boccoli G, Starnari R (2022). Opioid-free segmental thoracic spinal anesthesia with intrathecal sedation for breast and axillary surgery: report of four cases. Local Reg Anesth.

[66] Giampaolino P, Della Corte L, Mercorio A (2022). Laparoscopic gynecological surgery under minimally invasive anesthesia: a prospective cohort study. Updates Surg.

[67] Ullah MM, Kamal MM, Begum SA, Hassan AF, Islam MJ, Khan MS (2022). Effectiveness of segmental thoracic spinal anaesthesia in breast surgery: an observational study. J Shaheed Suhrawardy Med Coll.

[68] Aljuba YM Sr, Amro AM, Alkadi AT, Taamrah H, Hamamdh MG (2022). Thoracic segmental spinal anesthesia for emergency cholecystectomy: a case report. Cureus.

[69] Prima A, Hamdi T, Sitepu JF, Ghozali I, Lubis A (2023). Thoracic spinal anaesthesia for paediatric upper extremity surgery in limited-resource hospital: a case report. Ain Shams J Anesth.

[70] Paul A, Borkar A (2023). Anaesthetic management for mastectomy in a male with unilateral gynecomastia: the utilization of thoracic segmental spinal anaesthesia and erector spinae plane block. Cureus.

[71] Singhal G, Mathur BL, Mathur AK (2023). Efficacy and safety of segmental spinal anaesthesia in laparoscopic cholecystectomy: a prospective study. Indian J Clin Anaesth.

[72] Ghozali I, Danayati N (2023). Segmental thoracic spinal anesthesia (TSA) for open reduction with Internal Fixation (ORIF) surgery in right humeral fracture patients: a case report. MEDULA.

[73] Ghozali I, Hendroko HT, Rudyanto D, Fernanda MD (2023). A case report of Modified radical mastectomy (MRM) in a 59-year-old female patient with comorbid bronchiectasis using Thoracic segmental Spinal Anesthesia (TSA). MEDULA.

[74] Ghozali I, Hamdi T, Arisandi R, Rahmadilla AP (2022). Single-shot Spinal Anesthesia segmental Thoracic or Thoracic Spinal Anesthesia (TSA) for embolectomy in acute Limb ischemia (Ali) Stage IIB: a case report. MEDULA.

[75] Patel J, Ponnusamy K, Patel A (2023). Thoracic spinal anaesthesia in upper abdominal surgeries. Indian J Clin Anaesth.

[76] Venkatesh TK, Parthasarathy S (2023). Single-shot segmental thoracic spinal anesthesia for a giant lipoma of the back of the chest. India J Med specialities.

[77] Kokare M, Goasavi Y, Upasani S, Goyal B (2023). Benefits of segmental spinal anaesthesia in patients undergoing laproscopic cholecystectomy: a retrospective study. Sch J App Med Sci.

[78] Haloi P, Biswas R, Bora A (2023). Thoracic segmental spinal anesthesia for radical nephrectomy in a patient with amyotrophic lateral sclerosis-a case report. Ain Shams J Anesth.

[79] Chandra R, Misra G, Datta G (2023). Thoracic spinal anesthesia for laparoscopic cholecystectomy: an observational feasibility study. Cureus.

[80] Chandra R, Misra G, Langoo SA (2023). A prospective observational study on the effectiveness of segmental spinal anesthesia in patients posted for modified radical mastectomies. Ann Case Report.

[81] Nagar S, Lohia R, Saluja V, Gupta S (2023). Segmental spinal anaesthesia for routine surgeries: efficacy and safety in ASA 1 & 2 patients-a case series study. Eur J Cardiovasc Med.

[82] Basta B, Vailati D, Mori L, Magistro C, Marino G (2024). Thoracic segmental anesthesia for major laparoscopic abdominal surgery in a heart transplant recipient: a case report. Cureus.

[83] Vailati D, Bonvecchio E, Secco G, Magistro C, Basta B (2024). Neuraxial anesthesia for combined left nephrectomy and left hemicolectomy in a one-lung patient. Cureus.

[84] Yusmita D, Ghozali I, Rahmadilla AP (2024). Single-shot thoracic spinal anesthesia (TSA) in posterior segment VI of the liver abscess for laparoscopy: a case report. MEDULA.

[85] Sada F, Kavaja F, Hamza A, Ukperaj BM (2024). A 74-year-old man with severe comorbidities and successful abdominal aortic aneurysm repair with thoracic segmental spinal anesthesia: a case report. Am J Case Rep.

[86] Haloi P, Biswas R, Bora AK, Mahanta D, Choudhury D (2024). Thoracic segmental spinal anesthesia in kyphoplasty: a case series. Bali J Anesthesiol.

[87] Haloi P, Biswas R, Bora A, Devi S (2024). Efficacy of thoracic segmental spinal anaesthesia in percutaneous nephrolithotomy: a retrospective observational study. J Clin Diagn Res.

[88] Deshpande JP, Nath RR, Kulkarni PB, Atram S (2024). Thoracic segmental spinal anaesthesia a boon in challenging case scenarios: a case series. Indian J Clin Anaesth.

[89] Gujarkar Mahatme K, Deshmukh PU, Borkar A, Bankar NJ, Mahatme P (2024). A high index of awareness about the inherent complications of thoracic segmental spinal anesthesia: a case of mastectomy with bronchiectasis under thoracic segmental spinal anesthesia. Cureus.

[90] Goyal L, Gupta S, Nagar S, Bhargava V (2024). Feasibility, safety, and efficacy of segmental spinal anesthesia with predominantly isobaric levobupivacaine: a tertiary care hospital study. Int J Integr Health Sci.

[91] Thalji M, Tarayrah R, Ruzaygat A, Motawe D, Ibedo F (2024). Classic incisional hernia repair under awake thoracic combined spinal -epidural anesthesia in a geriatric patient with multiple co-morbidities. Int J Surg Case Rep.

[92] Awang MA, bin Mohamad Yusof MF, bin Abd Razak MH (2024). Segmental thoracic spinal anesthesia for abdominal surgery; a report of three cases. Anaesth pain intensive care.

[93] Gupta NA, Agarwal SO, Goyal GA (2024). Quality and efficacy of general anesthesia versus segmental thoracic spinal anesthesia in modified radical mastectomy surgery: a single-center observational study. Asian J Pharm Cl in Res.

[94] Boykov N, Ferdinandov D, Vasileva P, Yankov D, Burev S, Tanova R (2024). Thoracic spinal anesthesia with intrathecal sedation for lower back surgery: a retrospective cohort study. Front Med (Lausanne).

[95] Patel N, Makwana H, Gadhavi A, Gadhavi R (2024). A study of the efficacy of segmental spinal anesthesia in patients undergoing laparoscopic surgeries. Int J Pharm Clin Res.

[96] Bajracharya NR, Joshi P, Neupane B, Shrestha R, Milan KC, Parajuli B (2024). Thoracic segmental spinal anesthesia for laparoscopic cholecystectomy: a case report. J Inst Med Nepal.

[97] Haloi P (2024). Thoracic segmental spinal anesthesia for cesarean section in a parturient with atrial septal defect-a case report: TSSA in ASD patient for LSCS. Rev Soc Port Anestesiol.

[98] Raj PG, Avinash L, Bhagyashree AB, Kumar KR (2024). Layered and thoracic segmental spinal anaesthesia in patients with kyphoscoliosis for various surgeries: a case series. J Clin Diagn Res.

[99] Chandra R, Sonawane K (2024). Revolutionizing breast surgery: A case report on the excision of a large fibroadenoma suggestive of phyllodes tumor under thoracic spinal anesthesia. Cureus.

[100] Thakuria R, A S AJ, Kumar G, Talawar P (2024). Thoracic continuous spinal anesthesia in patients with destroyed lung resulted in favorable perioperative outcome. A A Pract.

[101] Saha SK, Saha H, Ullah MA, Ranjan R, Adhikary AB (2024). Awake thoracic surgery for severe pulmonary hypertension: a case report. Bangabandhu Sheikh Mujib Med Univ J.

[102] Aljuba YM, Alkadi AT, Hamamdh MG (2024). Segmental thoracic spinal anesthesia for critical patients undergoing abdominal surgeries: a case series and literature review. Cureus.

[103] Singhal G, Choudhary R, Choudhary P (2025). Comparison between general anesthesia and thoracic spinal anesthesia in total laparoscopic hysterectomy. Indian J Clin Anaesth.

